# HLA-DQ restricted activation of nitroso-sulfamethoxazole-specific CD4+ T-lymphocytes

**DOI:** 10.1186/2045-7022-4-S3-P110

**Published:** 2014-07-18

**Authors:** Monday Ogese, Katy Saide, Paul Whitaker, Daniel Peckham, Lee Faulkner, Ana Alfirevic, Munir Pirmohamed, Kevin Park, Dean Naisbitt

**Affiliations:** 1 University of Liverpool, MRC Centre for Drug Safety Science, UK; 2 Regional Adult Cystic Fibrosis Unit, St. James’s Hospital, UK

## Background

Sulfamethoxazole hypersensitivity has been used as a paradigm to study the role of drug metabolism in the activation of T-cells. CD4+ T-cells isolated from blood/skin of 100% of hypersensitive patients are activated with the protein-reactive metabolite nitroso sulfamethoxazole (SMX.NO). Since SMX.NO binds to multiple cellular proteins it is assumed that peptides derived from the modified protein interact with a number of diverse HLA molecules to activate T-cells; however, the HLA molecules that interact with SMX-NO modified peptides has not been studied.

## Method

The aims of this study were to examine the HLA molecules that present SMX.NO (derived peptides) to T-cells and determine the extent of alloreactivity. T-cell clones were generated from 4 hypersensitive patients with cystic fibrosis. Drug-specific proliferative responses and cytokine secretion were measured using [3H]-thymidine incorporation and ELISpot, respectively. Anti-human class I and class II (DR, DP, and DQ) antibodies were used to determine HLA restriction. Antigen presenting cells expressing different HLAs were used to define the alleles involved in the presentation of SMX.NO-derived antigens to T-cells.

## Results

A total of 976 clones were tested for SMX.NO reactivity. Forty nine CD4+ clones were activated to proliferate and secrete IFN-, IL-5, IL-13 and granzyme-B with SMX.NO. No cross reactivity with SMX was observed. The SMX.NO-specific response of clones was blocked with antibodies against MHC class II and HLA-DQ. Clones from 2 patients (Patient 1: HLA-DQB1*05:01:01G/ DQB1*06:03:01G; Patient 2: HLA-DQB1*02:01:01G/DQB1*02:01:01G) were used to define the DQ alleles involved in the presentation of SMX.NO derived antigens. SMX.NO-specific responses were detected with antigen presenting cells expressing HLA-DQB1*05 (patient 1) and HLA-DQB1*02 (patient 2), but not other HLA-DQB1 alleles.

## Conclusion

In conclusion, this study shows that HLA-DQ plays an important role in the activation of SMX.NO-specific T-cells from hypersensitive patients. T-cells from different patients recognized the drug antigen associated with different DQ alleles. However, clones from individual patients were highly DQ allele restricted.

